# Enhancing patient information texts in orthopaedics: How OpenAI's ‘ChatGPT’ can help

**DOI:** 10.1002/jeo2.70019

**Published:** 2024-09-17

**Authors:** Ali Yüce, Mustafa Yerli, Abdulhamit Misir, Murat Çakar

**Affiliations:** ^1^ Department of Orthopedic and Traumatology Prof. Dr. Cemil Taşcıoğlu City Hospital İstanbul Turkey; ^2^ Department of Orthopedic and Traumatology Göztepe Medical Park Hospital İstanbul Turkey

**Keywords:** arthroplasty, artificial intelligence, ChatGPT, healthcare information, hip, orthopaedic surgery, patient‐centered care, total hip replacement

## Abstract

**Purpose:**

The internet has become a primary source for patients seeking healthcare information, but the quality of online information, particularly in orthopaedics, often falls short. Orthopaedic surgeons now have the added responsibility of evaluating and guiding patients to credible online resources. This study aimed to assess ChatGPT's ability to identify deficiencies in patient information texts related to total hip arthroplasty websites and to evaluate its potential for enhancing the quality of these texts.

**Methods:**

In August 2023, 25 websites related to total hip arthroplasty were assessed using a standardized search on Google. Peer‐reviewed scientific articles, empty pages, dictionary definitions, and unrelated content were excluded. The remaining 10 websites were evaluated using the hip information scoring system (HISS). ChatGPT was then used to assess these texts, identify deficiencies and provide recommendations.

**Results:**

The mean HISS score of the websites was 9.5, indicating low to moderate quality. However, after implementing ChatGPT's suggested improvements, the score increased to 21.5, signifying excellent quality. ChatGPT's recommendations included using simpler language, adding FAQs, incorporating patient experiences, addressing cost and insurance issues, detailing preoperative and postoperative phases, including references, and emphasizing emotional and psychological support. The study demonstrates that ChatGPT can significantly enhance patient information quality.

**Conclusion:**

ChatGPT's role in elevating patient education regarding total hip arthroplasty is promising. This study sheds light on the potential of ChatGPT as an aid to orthopaedic surgeons in producing high‐quality patient information materials. Although it cannot replace human expertise, it offers a valuable means of enhancing the quality of healthcare information available online.

**Level of Evidence:**

Level IV.

**Abbreviations**
AI: artificial intelligence
ChatGPT: chat generative pretrained transformer
HISS: hip information scoring system

## INTRODUCTION

The widespread availability of information on the internet has led patients to seek healthcare information online, making it easily accessible [5]. Patients can search the internet using search engines or directories. The quality of information obtained in this capacity varies in terms of peer review and accuracy [1].

With the increased use of the internet, orthopaedic surgeons are now expected not only to provide medical information but also to explain and evaluate the information obtained online [1]. Therefore, recognizing existing web materials, recommending websites to patients, joining communities that provide high‐quality web‐based education, or creating their own websites with links to accurate patient information sources has become a necessity for orthopaedic surgeons [1, 14].

ChatGPT (chat generative pretrained transformer) is an artificial intelligence (AI) tool based on natural language processing, developed by OpenAI. ChatGPT is a chatbot‐based technology. A chatbot is a type of software that generates text similar to human‐like conversation [9]. This technology, capable of generating human‐like text, has been seen as a tool that can reduce the workload of writing scientific journal articles while meeting academic writing standards. Its potential has been demonstrated by being accepted as an author in multiple journals and even passing the United States Medical Board exams [8, 10, 17, 20, 21]. Additionally, ChatGPT can be used as an aid in medical education, research, and clinical management [2, 6, 12, 16, 24].

In the future, chatbots can assist orthopaedic surgeons in patient information and education. This can lead to the rapid and high‐quality preparation of websites containing more information and video text materials. The shortcomings that experts may overlook when preparing these texts can be filled in by chatbots, thereby enhancing the quality of the content. Therefore, in this study, we aimed to evaluate ChatGPT's ability to detect deficiencies in patient information and education related to total hip arthroplasty on websites and assess its recommendations for these deficiencies.

## METHODS

### Study design

On August 19, 2023, using Google Chrome (version 92.0.4515.159‐64 bit) with the browsing history cleared and cookies deleted, a standard query was conducted on the Google search engine using the term ‘total hip arthroplasty.’ The first 25 websites listed on the search results page were evaluated. During the evaluation, no priority was given to sponsored results, and websites were included only if they appeared in the top 25 results. This method was chosen because it has been used in previous studies assessing the quality of websites [4, 13].

### Inclusion and exclusion criteria

This study aimed to evaluate the content of the websites that patients encounter in the top rankings of their internet searches and that they might prefer to obtain information about their conditions. Therefore, peer‐reviewed scientific articles (five sites), menus and title pages lacking content (three sites), dictionary definitions (three sites), news and blogs (two sites), websites consisting solely of videos, websites unrelated to total hip arthroplasty (three sites), and websites with excessively long text that could not be evaluated by the chatbot were excluded. A total of 10 websites were included in this study (Table 1). Some of the included websites also had subheading links related to total hip arthroplasty, video presentations, and links to additional websites for education. Only the main texts related to total hip arthroplasty on these websites were evaluated. Videos, links to additional websites, and suggestions in separate windows under subheadings were not assessed.

**Table 1:** Internet addresses of the websites included in the study.

Website	Link
1	https://www.hss.edu/condition-list_hip-replacement.asp
2	https://www.mayoclinic.org/tests-procedures/hip-replacement/about/pac-20385042
3	https://www.hopkinsmedicine.org/health/treatment-tests-and-therapies/hip-replacement-surgery
4	https://www.nhs.uk/conditions/hip-replacement/
5	https://my.clevelandclinic.org/health/treatments/17102-hip-replacement
6	https://www.healthdirect.gov.au/hip-replacement
7	https://www.amplitude-ortho.com/en/total-hip-arthroplasty-tha
8	https://radiopaedia.org/articles/total-hip-arthroplasty
9	https://www.arthritis-health.com/surgery/hip-surgery/total-hip-replacement-surgical-procedure
10	https://www.stefankreuzermd.com/total-hip-replacement.html

### Hip information scoring system (HISS)

To evaluate the websites, the scoring system used by Köller et al. to determine the quality of YouTube videos was employed [2]. This scoring system was chosen because it had been previously used, and interobserver agreement was excellent. The websites included in the study were evaluated twice, one month apart, by two senior orthopaedic surgeons. As a result of these evaluations, the intraobserver ICC value for the first observer was calculated as 0.989, the intraobserver ICC value for the second observer was calculated as 0.978, and the interobserver ICC value was calculated as 0.793. In this scoring system, ‘diagnostic information’ and ‘treatment information’ were evaluated separately, each comprising a 12‐point scale. The quality of videos was categorized as low quality (0–3 points), medium quality (4–7 points), and excellent quality (8–12 points) out of a total of 12 points [13]. In this study, the ‘diagnostic information’ and ‘treatment information’ scoring systems were combined into a single scoring system, referred to as the Hip Information Scoring System (HISS), with a total score of 24 points (Table 2). The quality of web texts was categorized as low quality (0–8 points), medium quality (9–16 points), and excellent quality (17–24 points) out of a total of 24 points.

**Table 2:** Hip information scoring system (HISS).

Diagnostic information	Point
**Disease summary**	1
History	
Location of hip pain (groin, lateral, buttock, anterior thigh, knee)	1
Association of pain and activity or pain at night	1
Limitations with walking (i.e., reduced walking distance, limp, assisting devices)	1
Difficulties with activities of daily living: ascending stairs, rising from sitting, standing, bending to the floor, walking on a flat surface, getting in and out of car or on/off bus, going shopping, putting on your socks/stockings, rising from bed, taking off your socks/stockings, lying in bed, getting in and out of the bath, sitting, getting on/off the toilet, performance of heavy domestic duties, performing light domestic duties	2 (0.5 point each)
Stiffness (after waking in the morning, after sitting, resting in the day)	1
Primary, secondary arthritis (DDH, femoral/acetabular osteotomy, trauma, osteonecrosis, infection, FAI, Paget's disease)	1
**Physical exam**	
Limited range of motion (decreased flexion/internal rotation, flexion contracture, rotational contractures)	1
Leg length discrepancy	1
**Imaging**	
Conventional radiographs (AP pelvis, AP hip)	1
Advanced imaging if indicated (CT, MRI)	1
**Treatment information**	
**Conservative treatment**	
Patient education, NSAD, weight loss, activity modification (avoidance of high‐impact activities, usage of cane for a short period), hip injection, physical therapy (stretching, strengthening)	3 (1 point each)
**Surgical procedure**	
Explanation of the concept of total hip replacement or hip resurfacing	1
Approach to the hip (anterior, anterolateral, lateral, posterior)	1
Bearing surface (MoP, CoC, MoM, CoP)	1
Fixation (uncemented, cemented, hybrid)	1
Restoration of physiologic hip biomechanics (offset, leg length)	1
**Postoperative**	
Postoperative mobilization (hip precautions including no flex over 90°, adduction, internal rotation) and physiotherapy including rapid recovery	1
Outcome (improved function, pain‐free, improved quality of life)	1
**Complications**	
Infection, periprosthetic fracture, dislocation, nerve injury, vascular injury, venous thromboembolism, heterotopic ossification, pneumonia, leg length discrepancy, loosening	2 (0.5 point each)
Total	24

*Note*: Except for difficulties in daily living activities, one point was given for each item mentioned and 0.5 point was given for each activity mentioned. One point was given for each mentioned item except for the categories conservative treatment and complications, for which 0.5 points were given for each item mentioned.

Abbreviations: DDH, development dysplasia of hip; FAI, femoroacetabular impingement.

### ChatGPT and text evaluation

There are various publicly available and well‐known chatbot models on the market, with ChatGPT being the most popular among them [3, 18]. Therefore, assuming that orthopaedic surgeons can use a publicly accessible and easily reachable chatbot while preparing texts, in this study, ChatGPT‐4.0 (the version available to ‘ChatGPT Plus’ subscribers, 4 March 2023) was utilized.

Subsequently, the texts on the websites were individually uploaded to the ChatGPT‐4 chatbot. The chatbot was requested to evaluate the provided text, share its opinion on its quality, and enumerate any deficiencies in the text in terms of quality and educational value. The overall shortcomings identified by ChatGPT after its assessment, along with additional suggestions, were recorded. Then, the HISS scores were provided to ChatGPT‐4, asking it to evaluate these websites based on these scoring criteria and to report any missing criteria it identified.

Simultaneously, additional recommendations made by ChatGPT to enhance the quality of these websites were assessed. Furthermore, the HISS equivalent scores for the deficiencies identified by ChatGPT in the texts were determined. Thus, it was evaluated whether the quality of the text, according to HISS, could be improved if these deficiencies identified by ChatGPT were addressed and incorporated into the text.

### ChatGPT prompt

- For each text evaluation, a new chat session was initiated in ChatGPT. This was because when multiple text evaluations were requested in the same chat, ChatGPT considered the previously evaluated text in the same conversation.
- After initiating a new chat session, ChatGPT was posed the question, ‘I will provide you with a text. What are your thoughts regarding its educational value for patients? What are your suggestions to improve its quality?’
- After providing its recommendations on the text, ChatGPT was further asked, ‘I will give you some criteria. Can you inform me of the criteria that are not mentioned in the text?’
- ChatGPT provided an answer to this query, after which the text was presented.
- After the text, ChatGPT requested the criteria. The HISS criteria were presented to ChatGPT in a numbered list from 1 to 20. During this process, only the criteria were provided. The corresponding scores for the criteria were not presented to ChatGPT, nor was it asked to perform any scoring.
- ChatGPT documented the criteria that were missing in the text based on the HISS guidelines.

### Evaluation of texts

A different senior orthopaedic surgeon evaluated the web texts according to this scoring system, and the deficiencies in patient education on these websites were identified. In the texts, criteria missing according to HISS were recorded. This allowed for the evaluation of ChatGPT's success in identifying missing criteria in these texts. When the deficiencies detected by ChatGPT were added to the text, the new score the updated version of the text would receive according to HISS and the stage of its quality based on HISS were determined.

### Statistical analysis

The data obtained from the evaluations were analyzed using statistical methods to assess the intraobserver and interobserver reliability. Intraobserver reliability refers to the consistency of the same observer's assessments across multiple evaluations, while interobserver reliability measures the agreement between different observers. The intraobserver reliability was assessed by having each of the two senior orthopaedic surgeons evaluate the texts twice, with at least one week between the evaluations. The interobserver reliability was determined by comparing the scores assigned by the two different surgeons. The consistency of the evaluations was quantified using Cohen's kappa coefficient for categorical data and the intraclass correlation coefficient (ICC) for continuous data. Kappa values were interpreted as follows: <0.20 as poor agreement, 0.21–0.40 as fair, 0.41–0.60 as moderate, 0.61–0.80 as good, and >0.80 as excellent agreement. ICC values were interpreted similarly: <0.50 as poor, 0.50‐0.75 as moderate, 0.75–0.90 as good, and >0.90 as excellent. The statistical significance level was set at *p* < 0.05. All analyses were conducted using SPSS software (version 25.0; IBM Corp.).

## RESULTS

### General comments by ChatGPT on text deficiencies

When asked to review the texts without being given HISS criteria, ChatGPT provided the following common suggestions for improving the texts after its general assessment:

1. *Language and expression:* ChatGPT mentioned that technical terms in the texts can be confusing for patients. It recommended using simpler language for better understanding and suggested explaining these terms through illustrations to enhance comprehensibility.
2. *Frequently asked questions:* ChatGPT suggested that adding frequently asked questions and their answers to the texts could increase engagement.
3. *Patient experiences:* ChatGPT recommended incorporating quotes from real patients' experiences to make the text more personal and meaningful.
4. *Cost and insurance coverage:* ChatGPT noted that providing general information about the potential cost of the procedure for patients could be helpful, emphasizing the importance of cost and insurance coverage information.
5. *Preoperative and postoperative period:* ChatGPT highlighted the need to include modifications to reduce risks during the preoperative period and detailed information on rehabilitation and lifestyle changes during the postoperative period.
6. *References:* ChatGPT stated that adding references to indicate the sources of information could increase the text's credibility.
7. *Personalization:* ChatGPT emphasized that every patient is different and the procedures mentioned in the text represent a general approach. It suggested adding a warning or information encouraging patients to contact their doctors for personalized guidance.
8. *Emotional support and psychological preparation:* ChatGPT recommended adding information about the importance of emotional support and psychological preparation before and after surgery.
9. *Others:* ChatGPT identified specific deficiencies related to total hip arthroplasty in the texts. However, these identified deficiencies varied in scope and were generally part of a broader assessment.

### ChatGPT's success in detecting deficiencies according to HISS criteria

The total scores of the websites included in the study, as determined by the observer based on the HISS, along with their quality stages and score distribution among subgroups, are summarized in Table 3. In the rightmost two columns of Table 3, the corresponding HISS scores of the deficient criteria identified by ChatGPT‐4 are presented, and the potential new HISS quality stages that the websites would attain upon incorporating these identified deficiencies into the text are also indicated.

**Table 3:** Total hip information score system (HISS) scores of the texts after the observer's evaluation and the scores of the missing criteria determined by ChatGPT.

Website	Total HISS point after observer's evaluations	HISS quality according to observer's evaluations	Scores of missing criteria determined by ChatGPT	If the missing criteria specified by ChatGPT‐4 are added to the texts, the quality of the website according to HISS
**1**	14.5	Medium	5	Excellent
**2**	11	Medium	6	Excellent
**3**	11	Medium	7	Excellent
**4**	12.5	Medium	9.5	Excellent
**5**	11	Medium	13	Excellent
**6**	10	Medium	14	Excellent
**7**	4	Low	19	Excellent
**8**	7	Low	15	Excellent
**9**	3	Low	21	Excellent
**10**	11	Medium	11	Excellent

## DISCUSSION

The study's findings indicate that ChatGPT can enhance the quality of patient educational texts on ‘total hip arthroplasty.’ However, intraobserver and interobserver reliability were taken into account to ensure the consistency and accuracy of evaluations. Furthermore, it is noteworthy that in the literature, most of ChatGPT's applications in healthcare settings and medical writing and research consist of opinion pieces, comments, and reviews, with a minority being research articles [9, 15]. This study stands out as the first research article investigating the effectiveness of ChatGPT in improving the quality of patient education texts on ‘total hip arthroplasty.’

The interest in using chatbots in the field of education is on the rise. Chatbots are software programs that can engage in verbal or written conversations with human users and fulfil their requests using question‐and‐answer methods [11]. Since its launch, ChatGPT has found its place in higher education. This chatbot supports more than 40 languages and excels in generating human‐like dialogues [23]. The primary concerns regarding the use of ChatGPT in orthopaedic surgical research are creativity and judgement [21]. In the context of health education, chatbots can query existing information, promote higher student engagement in a learning task, or support higher‐level cognitive activities [22]. However, it is essential to note that while ChatGPT has the potential to revolutionize health education, AI technology cannot replace human expertise and judgement [7]. Nevertheless, it appears that ChatGPT can be beneficial in enhancing the content quality of texts prepared by orthopaedic surgeons. It can be used to rapidly identify deficiencies in patient information texts prepared by orthopaedic surgeons and improve their quality. This way, high‐quality texts can be prepared quickly and made available to patients. In the future, using chatbots for detecting overlooked deficiencies based on certain criteria in the preparation of patient information texts can save time. Additionally, high‐quality texts can be continuously provided to patients. Even a prepared text can be periodically evaluated by a chatbot to correct deficiencies with up‐to‐date information. This way, always current and high‐quality texts can be made available to patients. However, this is currently not possible. If a regular and up‐to‐date version of ChatGPT is made available, this situation will become usable for orthopaedic surgeons.

In a recent study evaluating ChatGPT's responses regarding hip osteoarthritis and total hip arthroplasty, it was noted that ChatGPT's responses were largely accurate, but they tended to be superficial due to the applied word limit [21]. Using ChatGPT in this manner, as shown in this study, could provide critical information to patients about their health and potential outcomes, thereby reducing anxiety and achieving better results. However, it was pointed out that in its current form, ChatGPT could potentially be a danger as a perioperative information source due to the word limit, which could lead to the omission of important references and recent research [21]. Considering this, it can be stated that for the time being, or at least soon, texts designed by orthopaedic surgeons will remain crucial in patient education. Additionally, the initial risk of a text prepared entirely by ChatGPT is also a concern. While ChatGPT cannot be used alone for patient education, it can assist in the preparation of high‐quality informative texts.

The ChatGPT database does not include data from the last few years [21]. This naturally hinders its ability to assess topics that evolve from day to day [21]. Although ChatGPT can significantly enhance the clarity and fluency of written material, maintaining human supervision throughout the process is very important. This is because AI can produce content that appears authoritative, but this content can be erroneous, incomplete, or biased. Incorrect GPT‐4 responses, known as ‘hallucinations,’ can be particularly harmful in the medical field. Therefore, it's important to check or verify the output of GPT‐4 [9]. Taking these into account, it's possible for the outdated ChatGPT to make incorrect assessments when evaluating the gaps in the text. Consequently, ChatGPT's ability to quickly prepare high‐quality texts for patients is limited and should be approached with caution.

Various scoring systems specific to orthopaedic diseases have been designed in the literature to evaluate the quality of information related to patient education [13, 25]. HISS is one of these scoring systems [13]. This study showed that intraobserver and interobserver reliability assessments using HISS were applicable. However, fundamentally, HISS only considers the pretreatment and posttreatment states of the disease. For orthopaedic surgeons, it is primarily important to have accurate and clear information about the disease. However, when looking at the deficiencies identified by ChatGPT in the texts, it emphasizes factors that affect the patient's perspective on the disease and treatment. This information is actually valuable for orthopaedic surgeons. No matter how high the information content quality of a text is, it is essential to remember that the reader is not a doctor. Ensuring the patient's perspective on total hip arthroplasty and alleviating their concerns is as important as providing accurate information for maintaining the patient–doctor relationship.

ChatGPT appears to have potential applications in various areas, including discovering new drugs, writing literature reviews, improving medical reports, providing medical information, enhancing research methods, analyzing data, and personalizing medication [19]. In a study where a word limit was imposed, ChatGPT's responses were largely accurate, but they tended to be superficial due to the word limit [21]. When the texts were evaluated by ChatGPT without HISS criteria, the focus was on deficiencies in general text characteristics, but after the criteria were provided, it succeeded in detecting most of these deficiencies. Additionally, two websites were excluded from the study because their texts were too long for ChatGPT to evaluate. The incapability of evaluating lengthy texts is a limitation of ChatGPT. This is because when the content quality of texts is intended to be enhanced, the length of the text will inevitably increase. However, the purpose of this study was to utilize ChatGPT versions that are readily available to everyone. The 4.0 model is one of these. This version was chosen as it serves as a tool that an orthopaedist preparing patient information can quickly access and use to reduce their workload. It should be remembered that when more advanced ChatGPT versions are used, there will be no text limitations.

One of the significant limitations of the study is that it examined text quality according to standard criteria. Considering that these texts focus more on information content, the understandability and engagement for patients are unknown. Also, as ChatGPT pointed out, personalization for individual patients was missing in these texts. Additionally, it is known that ChatGPT provides different responses to repeated questions, raising questions about whether the algorithm alters the literature it provides with each response [21]. In this study, ChatGPT's instant responses to the questions asked were evaluated. ChatGPT is a dynamic software capable of providing different responses based on the user, given criteria, and text characteristics. At the same time, hallucinations and ChatGPT not having up‐to‐date data are other factors that may affect ChatGPT's responses when evaluating texts [9, 21]. Therefore, whether ChatGPT's success in improving the quality of texts related to total hip arthroplasty varies according to the user, given criteria, and text characteristics remains an open question.

One of the limitations of this study was that the texts included in the study were not scored by ChatGPT using the HISS scoring system, and the agreement of the obtained scores with other observers was not evaluated. However, in a recent study where YouTube video texts related to rotator cuff were evaluated using a specific scoring system for ChatGPT, it was reported that ChatGPT might currently be insufficient in this area [26]. Therefore, the design of this study focused on ChatGPT's ability to detect information deficiencies. Lastly, the small number of websites included in this study is a limitation. More extensive studies including a larger number of websites are needed.

## CONCLUSIONS

ChatGPT can potentially be a useful tool for orthopaedic surgeons in enhancing the quality of patient information forms prepared for total hip arthroplasty. It seems that it could be a valuable tool in designing and delivering high‐quality websites more quickly in the future. However, it is crucial to note that for the time being, or at least soon, texts designed by orthopaedic surgeons will remain essential in patient education. Although ChatGPT has the potential to revolutionize patient education, it is essential to emphasize that AI technology cannot replace human expertise and judgement.

## AUTHOR CONTRIBUTIONS

All authors contributed to the study conception and design. Ali Yüce, Mustafa Yerli, Abdulhamit Misir and Murat Çakar performed the data collection and analysis and prepared the material. Ali Yüce prepared the first draft of the manuscript and all authors commented on previous versions of the manuscript. All authors read and approved the final manuscript.

## CONFLICT OF INTEREST STATEMENT

The authors declare no conflict of interest.

## ETHICS STATEMENT

Ethical approval is not applicable for this article.

## References

[1] Beall 3rd, M.S. , Beall Jr. MS , Greenfield, M.L. & Biermann, J.S. (2002) Patient Internet use in a community outpatient orthopaedic practice. The Iowa Orthopaedic Journal, 22, 103–107. 12180601 PMC1888381

[2] Chatterjee, S. , Bhattacharya, M. , Pal, S. , Lee, S.‐S. & Chakraborty, C. (2023) ChatGPT and large language models in orthopedics: from education and surgery to research. Journal of Experimental Orthopaedics, 10, 128. Available from: 10.1186/s40634-023-00700-1 38038796 PMC10692045

[3] Coello, C.E.A. , Alimam, M.N. & Kouatly, R. (2024) Effectiveness of ChatGPT in coding: a comparative analysis of popular large language models. Digital, 4, 114–125. Available from: 10.3390/digital4010005

[4] Dy, C.J. , Taylor, S.A. , Patel, R.M. , Kitay, A. , Roberts, T.R. & Daluiski, A. (2012) The effect of search term on the quality and accuracy of online information regarding distal radius fractures. The Journal of Hand Surgery, 37, 1881–1887. Available from: 10.1016/j.jhsa.2012.05.021 22857909

[5] Fabricant, P.D. , Dy, C.J. , Patel, R.M. , Blanco, J.S. & Doyle, S.M. (2013) Internet search term affects the quality and accuracy of online information about developmental hip dysplasia. Journal of Pediatric Orthopaedics, 33, 361–365. Available from: 10.1097/BPO.0b013e31827d0dd2 23653022

[6] Fayed, A.M. , Mansur, N.S.B. , de Carvalho, K.A. , Behrens, A. , D'Hooghe, P. & de Cesar Netto, C. (2023) Artificial intelligence and ChatGPT in orthopaedics and sports medicine. Journal of Experimental Orthopaedics, 10, 74. Available from: 10.1186/s40634-023-00642-8 37493985 PMC10371934

[7] Feng, S. & Shen, Y. (2023) ChatGPT and the future of medical education. Academic Medicine, 98, 867–868. Available from: 10.1097/ACM.0000000000005242 37162219

[8] Flanagin, A. , Bibbins‐Domingo, K. , Berkwits, M. & Christiansen, S.L. (2023) Nonhuman “authors” and implications for the integrity of scientific publication and medical knowledge. Journal of the American Medical Association, 329, 637–639. Available from: 10.1001/jama.2023.1344 36719674

[9] Garg, R.K. , Urs, V.L. , Agrawal, A.A. , Chaudhary, S.K. , Paliwal, V. & Kar, S.K. (2023) Exploring the role of ChatGPT in patient care (diagnosis and treatment) and medical research: a systematic review. Health Promotion Perspectives, 13, 183–191. Available from: 10.34172/hpp.2023.22 37808939 PMC10558973

[10] Gwak, G. , Hwang, U. , Jung, S. & Kim, J. (2023) Search for medical information and treatment options for musculoskeletal disorders through an artificial intelligence chatbot: focusing on shoulder impingement syndrome. Journal of Musculoskeletal Science and Technology, 7, 8–16. Available from: 10.29273/jmst.2023.7.1.8

[11] Han, J.W. , Park, J. & Lee, H. (2022) Analysis of the effect of an artificial intelligence chatbot educational program on non‐face‐to‐face classes: a quasi‐experimental study. BMC Medical Education, 22, 830. Available from: 10.1186/s12909-022-03898-3 36457086 PMC9713176

[12] Khan, R.A. , Jawaid, M. , Khan, A.R. & Sajjad, M. (2023) ChatGPT—reshaping medical education and clinical management. Pakistan Journal of Medical Sciences, 39, 605–607. Available from: 10.12669/pjms.39.2.7653 36950398 PMC10025693

[13] Koller, U. , Waldstein, W. , Schatz, K.D. & Windhager, R. (2016) YouTube provides irrelevant information for the diagnosis and treatment of hip arthritis. International Orthopaedics, 40, 1995–2002. Available from: 10.1007/s00264-016-3174-7 27029480

[14] Krempec, J. , Hall, J. & Biermann, J.S. (2003) Internet use by patients in orthopaedic surgery. The Iowa Orthopaedic Journal, 23, 80–82. 14575255 PMC1888386

[15] Levin, G. , Meyer, R. , Yasmeen, A. , Yang, B. , Guigue, P.A. , Bar‐Noy, T. et al. (2023) Chat generative pre‐trained transformer‐written obstetrics and gynecology abstracts fool practitioners. American Journal of Oobstetrics & Gynecology MFM, 5, 100993. Available from: 10.1016/j.ajogmf.2023.100993 37127209

[16] Lower, K. , Seth, I. , Lim, B. & Seth, N. (2023) ChatGPT‐4: transforming medical education and addressing clinical exposure challenges in the post‐pandemic era. Indian Journal of Orthopaedics, 57, 1527–1544. Available from: 10.1007/s43465-023-00967-7 37609022 PMC10442004

[17] O'Connor, S. & ChatGPT (2023) Open artificial intelligence platforms in nursing education: tools for academic progress or abuse? Nurse Education in Practice, 66, 103537. Available from: 10.1016/j.nepr.2022.103537 36549229

[18] Pushpanathan, K. , Lim, Z.W. , Er Yew, S.M. , Chen, D.Z. , Hui'En Lin, H.A. , Lin Goh, J.H. et al. (2023) Popular large language model chatbots’ accuracy, comprehensiveness, and self‐awareness in answering ocular symptom queries. iScience, 26, 108163. Available from: 10.1016/j.isci.2023.108163 37915603 PMC10616302

[19] Ruksakulpiwat, S. , Kumar, A. & Ajibade, A. (2023) Using ChatGPT in medical research: current status and future directions. Journal of Multidisciplinary Healthcare, 16, 1513–1520. Available from: 10.2147/JMDH.S413470 37274428 PMC10239248

[20] Seth, I. , Rodwell, A. , Bulloch, G. & Seth, N. (2023) Exploring the role of open artificial intelligence platform on surgical management of knee osteoarthritis: a case study of ChatGPT. J Clin Cases Rep S, 13, 217–222. Available from: 10.46619/joccr.2023.6-S13.1075

[21] Seth, I. , Rodwell, A. , Tso, R. , Valles, J. , Bulloch, G. & Seth, N. (2023) A conversation with an open artificial intelligence platform on osteoarthritis of the hip and treatment. Journal of Orthopaedics and Sports Medicine, 05, 112–120. Available from: 10.26502/josm.511500088

[22] Stathakarou, N. , Nifakos, S. , Karlgren, K. , Konstantinidis, S.T. , Bamidis, P.D. , Pattichis, C.S. et al. (2020) Students’ perceptions on Chatbots’ potential and design characteristics in healthcare education. Studies in Health Technology and Informatics, 272, 209–212. Available from: 10.3233/SHTI200531 32604638

[23] van de Ridder, J.M.M. , Shoja, M.M. & Rajput, V. (2023) Finding the place of ChatGPT in medical education. Academic Medicine, 98, 867. Available from: 10.1097/ACM.0000000000005254 37162206

[24] Yüce, A. , Erkurt, N. , Yerli, M. & Misir, A. (2024) The potential of ChatGPT for high‐quality information in patient education for sports surgery. Cureus, 16, e58874. Available from: 10.7759/cureus.58874 38800159 PMC11116739

[25] Yüce, A. , Gür, V. , Yerli, M. & Misir, A. (2024) The lack of high‐quality educational resources about adhesive capsulitis on YouTube. Revista Brasileira de Ortopedia, 59, 260–268. Available from: 10.1055/s-0044-1785465 PMC11006515 38606132

[26] Yüce, A. , Yerli, M. & Mısır, A. (2024) Can Chat‐GPT assist the orthopedic surgeons in evaluating the quality of rotator cuff surgery patient information videos? Journal of Shoulder and Elbow Surgery. In press. 10.1016/j.jse.2024.04.021 38852711

